# Predicting global educational inequality with a hierarchical belief rule base model

**DOI:** 10.1038/s41598-025-97390-9

**Published:** 2025-04-11

**Authors:** Aosen Gong, Wei He, Gaixia Ge, Cuiping Yang, Shaohua Li

**Affiliations:** 1 School of Computer Science and Information Engineering, Harbin Normal University, Harbin, China; 2 Harbin Donghu Road School, Harbin, China; 3 Dalian University of Foreign Languages, Dalian, China

**Keywords:** Education, Belief rule base, Hierarchical structure, Interpretability, Sustainability education, Computer science, Sustainability

## Abstract

Global educational inequality is influenced by socio-economic development, especially in low-income and conflict-affected regions. Predicting such inequality allows researchers and policymakers to design better education policies and resource allocation strategies. The Belief Rule Base (BRB) is used as an interpretable model that incorporates expert knowledge, making it suitable for these predictions. However, BRBs face challenges, including rule explosion due to excessive feature information and a lack of standardized hierarchical modeling. In addition, parameters in optimization processes are often influenced by randomness, causing them to deviate from expert knowledge, which reduces interpretability. To address these issues, we adopt the interpretable hierarchical confidence rule base (HBRB-I) model, which enables self-organized construction of hierarchical structures through a multilayer tree structure (MTS), and a new optimization scheme to improve accuracy while retaining interpretability. Experimental results show that the HBRB-I model achieves high accuracy and robustness in predicting global educational inequality, which also provides data support for equitable and sustainable distribution of global educational resources. The interpretability of the model allows policymakers to develop forward-looking education policies that are aligned with sustainable development goals.

## Introduction

Education is the cornerstone of sustainable social development, improving the quality of life of individuals and driving economic growth, social stability and environmental stewardship. However, educational resources are unequally distributed globally, particularly in low-income and conflict-affected regions. Interpretable forecasting models can accurately assess this inequality, providing researchers and policymakers with transparent insights into the education landscape. Unlike traditional ‘black box’ models, interpretable models clarify how factors influence educational outcomes, allowing resource allocation and policy decisions to be justified. This transparency is essential for promoting global educational equity and sustainable development, enabling targeted interventions that address both current and future inequalities. Predictive models also act as early warning systems, identifying potential problems of educational inequality and allowing for timely action. Predicting educational inequality is therefore essential for promoting global equity and sustainable development^1–3^.

Educational inequality can be more accurately assessed through interpretable predictive models, providing researchers and policy makers with transparent insights into the educational landscape. Based on the interpretability of the models, existing predictive models can be classified into three categories: black-box, white-box and grey-box models. Black-box models are typically characterized by their ability to handle complex data relationships and provide high prediction accuracy, as seen in models such as Random Forests (RFs)^4,5^, Support Vector Machines (SVMs)^6^, and Deep Neural Networks (DNNs)^7,8^. The internal algorithms of these models are designed with a high degree of complexity, often involving many parameters and non-linear relationships that are invisible to the user. This “black box” nature makes it difficult to explain the specific decision-making process when the model makes a prediction. For example, when a country’s educational inequality index is predicted by a black-box model, policymakers are not provided with a clear understanding of the factors at play, nor is the exact relationship between the model’s outputs and input variables made apparent. This lack of interpretability has limited the use of these models in the social sciences, particularly in areas related to public policy and resource allocation. White-box models, such as linear regression and simple decision trees^9,10^, are highly transparent. The effect of each input variable on the output can be directly observed, which facilitates understanding and trust, especially in domains such as educational policy. However, white-box models assume simple relationships and cannot effectively capture complex, non-linear interactions. Gray box models aim to balance interpretability and accuracy. These models, including extended decision trees or semi-supervised models that incorporate expert knowledge, allow partial visualization of decision paths while preserving complex mechanisms. Although they offer better interpretability than black-box models, challenges remain as their interpretability is not always intuitive and prediction accuracy may not improve significantly over black-box models^11–13^.

To address the lack of balance between accuracy and interpretability in traditional models, the Belief Rule Base (BRB) model was developed as an interpretable expert system that combines a data-driven approach with expert knowledge, using transparent evidential reasoning (ER) as its engine^14–17^. Unlike black-box models, the reasoning process and each decision node of a belief rule base model are made interpretable, allowing users to clearly understand how predictions are made and to trace the logic behind each decision. This interpretability has led to the widespread use of BRBs in fault diagnosis, health monitoring, and risk assessment. The BRB consists of a rule base and an inference engine, where the rule base contains the rules and the attributes within them. The rules are formed by the Cartesian product of the reference values of the input attributes. For example, in a global education analysis, three attributes are considered: the latitude and longitude of the region, the economic capacity of the region, and the population size of the region. Assigning three values to each attribute generates 27 rules. As the input attributes are expanded, the number of rules grows exponentially, leading to what is known as rule explosion. To address the problem of combinatorial rule explosion, researchers have attempted to minimize the number of features and thereby reduce the number of rules generated by applying feature engineering techniques such as PCA or LDA for dimensionality reduction^18–20^. However, this approach significantly compromises the interpretability of the feature data itself, resulting in a loss of model interpretability. Recent studies have developed the Extended BRB (EBRB) by expanding the description of the rule’s antecedent, which can effectively reduce combinatorial rule explosion, but this expansion introduces more knowledge into each rule, thereby affecting the readability of the model^21,22^. The Approximate BRB (ABRB) model has been proposed, which constructs single-input–output belief rules and implements an online updating BRB system to solve the combinatorial rule explosion problem to some extent^23^. However, in the educational domain, BRB-based models are often associated with breakdowns in interpretability due to optimization, a problem that becomes more apparent in the online updating of BRBs. In particular, the parameters of BRBs, which are typically constituted by expert knowledge in the domain, are prone to deviate from this expert knowledge or subjective awareness during the optimization process due to the stochastic nature of optimization algorithms, thereby undermining the interpretability of the model^24–26^.

To address the problems, this paper constructs a hierarchically structured interpretable BRB (HBRB-I), which consists of multiple sub-BRB models to reduce the number of rules. At the same time, a general modeling rule has been designed for the rational construction of sub-BRBs at each level. To solve the problem of optimized interpretability, an interpretable optimization method applicable to hierarchically structured BRBs has been designed, along with the introduction of a new reference value optimization scheme. This approach aims to improve model accuracy while maintaining model interpretability.

The paper is organized as follows. Section “BRB basics and problem description” describes the basic rules of BRB and the challenges of predicting global educational inequalities. The modeling strategy for the HBRB-I model is presented in Section “Modeling process of HBRB-I based on multilayer tree structure”. Section “Interpretable optimization algorithm construction” discusses the interpretable optimization strategy. The validation of the model and the optimization algorithm through case studies is conducted in Section “Case study”. Finally, the research results of this paper are summarized in Section “Conclusions”.

## BRB basics and problem description

### Rule description of BRB

The rules in BRB are confidence rules generated based on IF–THEN, which include a premise part consisting of antecedent attributes and a result part consisting of consequent attributes, and the *k*th rule, for example, is represented as follows:

1 $$\begin{gathered} R_{k} : \, IF{ (}x_{1} \, is \, A_{1}^{k} ) \wedge (x_{2} \, is \, A_{2}^{k} {) } \wedge \cdots \wedge (x_{M} \, is \, A_{M}^{k} ) \hfill \\ \quad \quad \;THEN \, \{ (D_{1} ,\beta_{1,k} ),(D_{2} ,\beta_{2,k} ) \cdots (D_{N} ,\beta_{N,k} )\} \left( {\sum\limits_{n = 1}^{N} {\beta_{N,k} } \le 1} \right), \hfill \\ \quad \quad \;WITH \, rule \, weight \, \theta_{k} \hfill \\ \quad \quad \;AND \, attribute \, weights \, \delta_{i} \hfill \\ \end{gathered}$$

where $A_{i}^{k}$ denotes the reference value about the sampled data $x_{i} (i = 1, \ldots ,M)$ and $\beta_{n,k} (n = 1, \ldots ,N;k = 1, \ldots ,L)$ denotes the belief degree about the posterior attribute $D_{n}$. $\theta_{k}$ is the weight of the *k*th belief rule. $\delta_{i}$ is the weight of attribute $X_{i}$. *L* is the number of rules in the belief rule base.

Taking the example of fault diagnosis of certain equipment, three corresponding input features are provided for sample *x*: {$A_{1}$: speed, $A_{2}$: voltage, $A_{3}$: temperature}. Each input feature is assigned three reference values, which are low, medium and high. The corresponding error states are given as {$D_{1}$: normal, $D_{2}$: Bad,$D_{3}$: Damaged}. The kth rule can then be interpreted as follows: if the speed is high, the voltage is high, and the temperature is high, then the fault state is represented as {$D_{1}$: 0, $D_{2}$: 0, $D_{3}$: 1}.

### Description of the problem

*Problem 1*: How to construct an interpretable predictive model of global education disparities? While traditional machine learning models are often not transparent and interpretable, BRB models are constructed by combining expert knowledge with easy-to-understand IF–THEN rules, making them interpretable. However, time series data are often used as input in global education forecasting. For example, trends in educational attainment between 2013 and 2020 need to be analyzed by the model, which requires it to handle continuous data over multiple points in time. When the BRB model is applied to time series data, the characteristics of each time point are treated as separate input dimensions. This results in an exponential growth in the number of rules as the model deals with multiple years of time series data, leading to the so-called rule explosion problem. Rule explosion not only increases the computational and storage complexity of the model but also makes real-time inference difficult to achieve, thereby reducing the practical value of the model in predicting global education levels.

2 $$HBRB - I = \left\{ {Sub - BRB^{1} ,Sub - BRB^{2} , \ldots ,Sub - BRB^{H} } \right\}$$

where *HBRB* is a type of BRB that uses a hierarchical structure and *Sub-BRB* is a *Sub-BRB* of one of the layers in the *HBRB*.

*Problem 2*: Interpretability of the Optimization Algorithm. The core strength of the BRB model lies in its parameters, which are typically determined by expert knowledge, ensuring that the model’s prediction process remains highly interpretable. In practice, however, it is often necessary to optimize these parameters to improve the model’s prediction accuracy. However, traditional optimization algorithms introduce a degree of randomness into the parameter adjustment process, which can disrupt the parameter structure established by the original expert knowledge. As a result, the interpretability of the model may be lost after optimization. When the internal parameters of the model no longer match the intuition and experience of the experts, the prediction results, although potentially more accurate, lead to a decision-making process that becomes ambiguous and difficult for users to understand. This loss of interpretability is particularly pronounced in sensitive areas such as predicting global educational attainment, where decision-makers require not only accurate predictions but also a clear understanding of the model’s reasoning logic to make informed policy judgments^24^.

3 $$\begin{gathered} {\text{Interpretability constraints:}}\left\{ {{\text{C|C}}_{1} {\text{,C}}_{2} {,} \ldots {\text{,C}}_{z} } \right\} \hfill \\ \Omega = optimization(data,E,C) \hfill \\ \end{gathered}$$

where *C* denotes interpretability constraints, $\Omega$ denotes optimized parameters, $optimization \, ( \cdot )$ denotes optimization algorithm, *E* denotes expert knowledge and *data* denotes sample data.

## Modeling process of HBRB-I based on multilayer tree structure

The modeling methodology for the HBRB-I model based on the multilayer tree structure (MTS) is given in Section “HBRB-I model construction based on MTS”. The inference process for HBRB-I is described in Section “The reasoning process of HBRB-I”, and the interpretability is given in Section “Interpretable definition of HBRB-I”.

### HBRB-I model construction based on MTS

In traditional HBRB construction methods, researchers typically rely on subjective judgment to define the input features for each BRB layer. This approach tends to manually select input features and define the hierarchy based on expert experience or intuitive understanding of the data. However, this experience-based approach can introduce bias and inconsistency in the construction process, especially when dealing with high-dimensional and complex datasets. Fixed feature selection and hierarchies may not be able to adapt to different datasets or scenarios, limiting the flexibility and adaptability of the model. In addition, due to the lack of a dynamic adjustment mechanism, traditional methods are difficult to effectively capture the non-linear relationships between attributes and potential dependencies in the hierarchical structure, which affects the generalization ability and accuracy of the model.

To solve this problem, this study adopts the multi-tier tree structure (MTS) method for hierarchical BRB construction. By introducing a hierarchical tree structure, MTS can automatically adjust the delineation and clustering of features at different levels according to the intrinsic characteristics of the data, thus avoiding the problem of relying on subjective judgments in the traditional methods^27,28^.

**Fig. 1:**
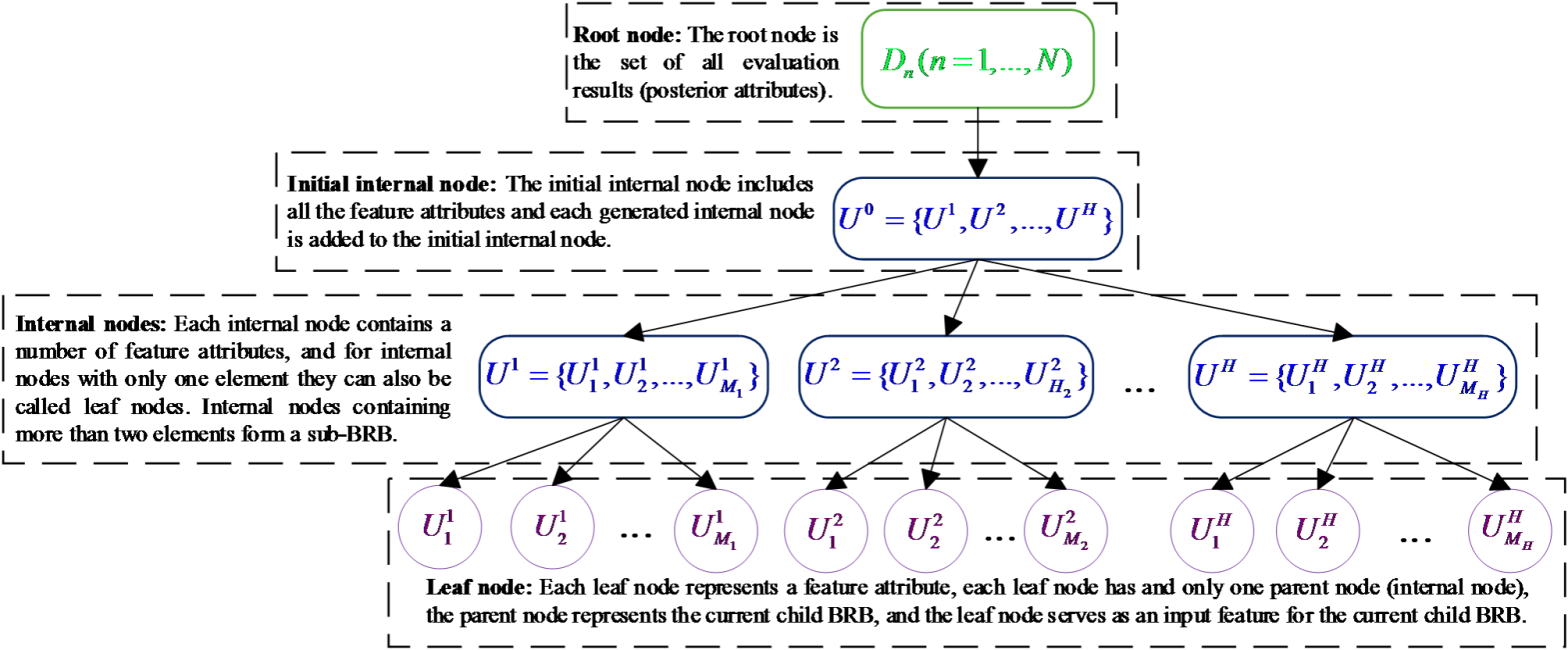
Structural diagram of a multilayer tree structure.

Specifically, MTS consists of three types of nodes: root nodes, internal nodes and leaf nodes. Root nodes: nodes that have no parent nodes become root nodes, which contain a set consisting of all evaluation results $D_{n} (n = 1, \ldots ,N)$. Internal nodes: nodes that have both root nodes and child nodes become internal nodes, and each internal node can be understood as a child BRB that contains the next level of leaf nodes or internal nodes. Leaf nodes: nodes that have no child nodes become leaf nodes, and each leaf node corresponds to a feature attribute. The construction of MTS is divided into three parts: defining the root node, defining the leaf nodes and hierarchical clustering. The structure is shown in Fig. 1 and the construction of MTS is described as follows:

*Step 1 (Define the root node)*: In a tree structure, the root node $U^{D}$ is the starting node of the tree, while in MTS, the root node is the node that contains all the descendant attributes $D_{n} (n = 1, \ldots ,N)$. At the same time, it also has the characteristics of tree structure: there is only one root node in MTS, which is the top-level node of MTS; the root node is the only node with no parent node.

*Step 2 (Define the leaf node)*: A leaf node $U_{m} (m = 1, \ldots ,M)$ is described as a collection of antecedent attributes (characteristic attributes). Were $U_{m} (m = 1, \ldots ,M)$ denotes an element in a leaf node, and each $U_{m}$ corresponds to an antecedent attribute (feature).

*Step 3 (Hierarchical clustering)*: Hierarchical clustering is the process of aggregating all leaf nodes (feature attributes) in some way into internal nodes, each of which contains at least one child node. The process of hierarchical clustering is shown below:

*Step 3.1 (Constructing the initial internal node)*: The initial internal node is the first internal node in the MTS, as shown in Fig. 1, and is fixed at the next level below the root node. The initial internal node contains the union of all the remaining internal nodes, i.e., $U^{0} = \{ U^{1} ,U^{2} , \ldots ,U^{H} \}$, where $U^{h} = \{ U_{1}^{h} , \ldots ,U_{{M_{h} }}^{h} \} (h = 1, \ldots ,H)$ denotes the *h*th internal node, which contains $M_{h}$ feature attributes.

*Step 3.2 (Calculate the relation value between two attributes (AR))*: After determining the initial internal nodes, the next step is to generate all internal nodes, for internal nodes other than the initial internal nodes, the aggregation between each node is performed by attribute correlation, so the correlation between each attribute needs to be calculated first. The calculation method is shown below:

4 $$\begin{gathered} AR(U_{m} ,U_{n} ) = \frac{{I(U_{m} ,U_{n} )}}{{H(U_{m} ,U_{n} )}} \hfill \\ H(U_{m} ,U_{n} ) = - \sum\limits_{m = 1}^{M} {\sum\limits_{n = 1}^{M} {P(m,n) \times \log P(m,n)} } \hfill \\ H(U_{m} ) = - \sum\limits_{m = 1}^{M} {P(m) \times \log P(m)} ,H(U_{n} ) = - \sum\limits_{n = 1}^{M} {P(n) \times \log P(n)} \hfill \\ I(U_{m} ;U_{n} ) = H(U_{m} ) + H(U_{n} ) - H(U_{m} ,U_{n} ) \hfill \\ m,n = 1, \ldots ,M \hfill \\ \end{gathered}$$

where $AR(U_{m} ,U_{n} )$ denotes the correlation between attribute $U_{m}$ and attribute $U_{n}$, which is generated by the ratio of mutual information $I(U_{m} ,U_{n} )$ and entropy $H(U_{m} ,U_{n} )$ between $U_{m}$ and $U_{n}$. *P(m)* is the probability that the attribute $U_{m}$ is equal to *m* and *P(n)* is the probability that the attribute $U_{n}$ is equal to *n*, and *T* denotes the number of data.

*Step 3.3 (Specify the size of the internal node)*: According to the attribute correlation calculation method given in Step 3.2, the correlation between all attributes can be calculated. To facilitate the construction of the tree structure, the number of elements in each internal node cannot exceed the number of elements $\varepsilon$, where $\varepsilon$ is the size of the internal node given by the researcher according to the actual situation.

*Step 3.4 (Constructing internal nodes)*: According to the AR value calculated in Step 3.2, each attribute should generate AR values with all attributes except itself, and further calculate the sum of AR values of each attribute with other attributes denoted as $Count_{i} = \sum\nolimits_{{U_{m} }}^{{U^{0} }} {AR(U_{i} ,U_{m} )} ,i = 1, \ldots M,$, where $Count_{i}$ denotes the sum of AR values of the *i*th attribute. Based on the internal node size $\varepsilon$ determined in Step3.3, the $\varepsilon$ elements with the smallest sum are first selected, and the selected elements are called master elements, each of which forms a separate internal node. The calculation method is shown below:

5 $$P_{c} = \left\{ \begin{gathered} \arg \min_{{U_{c} \in U^{h} }} \left\{ {\sum\limits_{{U_{i} }}^{{U^{h} }} {AR(U_{c} ,U_{i} )} } \right\},(c = 1, \ldots ,\varepsilon )\quad \quad \quad if\;\varepsilon = 1 \hfill \\ \arg \min_{{U_{c} \in U^{h} - \{ P_{1} , \ldots ,P_{c - 1} \} }} \left\{ {\sum\limits_{{U_{i} }}^{{\{ P_{1} , \ldots ,P_{c - 1} \} }} {AR(U_{c} ,U_{i} )} } \right\},\quad otherwise \hfill \\ \end{gathered} \right.$$

where $P_{c}$ denotes the *c*th main element. $U^{h} (h = 1, \ldots ,H)$ denotes the *h*th internal node. $\{ P_{1} , \ldots ,P_{c - 1} \}$ denotes the $P_{c - 1}$ th main element that has been selected. Only one master element is possible in each internal node. After s master elements are selected, s internal nodes are constructed accordingly, denoted as $\{ U^{1} = \{ P_{1} \} ,U^{2} = \{ P_{2} \} , \ldots ,U^{\varepsilon } = \{ P_{\varepsilon } \} \}$.

After that, all the remaining elements are added to the internal node where the main element is located in order according to the relationship between the AR values with the $\varepsilon$ main elements from the largest to the smallest, which is calculated as shown below:

6 $$\begin{gathered} U^{h} = \{ U_{m} \} \cup U^{h} (U_{m} \ne P_{c} ), \hfill \\ h = \arg \max_{c = 1,...,\varepsilon } \{ AR(P_{c} ,U_{m} )\} \hfill \\ \end{gathered}$$

For easy understanding, three internal nodes $U^{0} = \{ U^{1} = \{ X\} ,U^{2} = \{ Y\} ,U^{3} = \{ Z\} ,G\}$ are created in the dummy $U^{0}$, where each node contains only one main element, which are* X*, *Y*, and *Z*. At this point, there is still one element left that has not been added to the node as *G*. Based on the relationship between the AR value of element *G* and each main element, it is determined to which node *G* should be added, assuming that the AR value is denoted as $AR\left( {X,{\text{ G}}} \right) > AR\left( {Y,{\text{ G}}} \right) > AR\left( {Z,{\text{ G}}} \right)$. Element *G* has the highest correlation with element *X*. Therefore, element *G* is added to element *X* to form the set $U^{1} = \{ X,G\}$. And so on until all elements are aggregated into the corresponding set.

*Step 3.5 (Internal node optimization)*: After adding all attributes to the internal node where the main element is in Step 3.4, there may be a situation where the size of the set where a certain main element is located exceeds the specified internal node size $\varepsilon$. If the size of node $U^{h}$ exceeds $\varepsilon$, then for all elements in this set, recalculate the AR values of the attributes in $U^{h}$ with respect to each other according to Step 3.2 and re-cluster them according to Steps 3.3–3.4 to generate the next level of internal nodes $Sub - U^{h}$.

For example, the set $U^{h}$ contains the elements{*V*,*W*,*X*,*Y*,*Z*}, and assuming $\varepsilon = 2$, the size of $U^{h}$ exceeds the specified threshold , then $U^{h}$ is reclustered, and the AR between all attributes contained therein are computed, and reclustered into {*V*,*W*}, {*X*, *Y*}, and {*Z*} according to Step3.3-Step3.4. By analogy, recursively determine whether the size of each newly generated internal node satisfies the threshold , and reconstruct the internal nodes that do not satisfy it until all attributes are added to the unique internal node and the size of all internal nodes satisfy the given threshold $\varepsilon$.

*Step 3.6 (Generate Leaf Nodes)*: After recursively generating all internal nodes, leaf nodes are generated among all internal nodes that satisfy the size. Here there are two cases for the internal node $U^{h}$: (1) There is only one element in $U^{h}$, then $U^{h}$ can be directly turned into a leaf node. (2) If there are multiple elements in $U^{h}$ and the number of elements does not exceed $\varepsilon$, then each element in $U^{h}$ can generate its own leaf node.

**Fig. 2:**
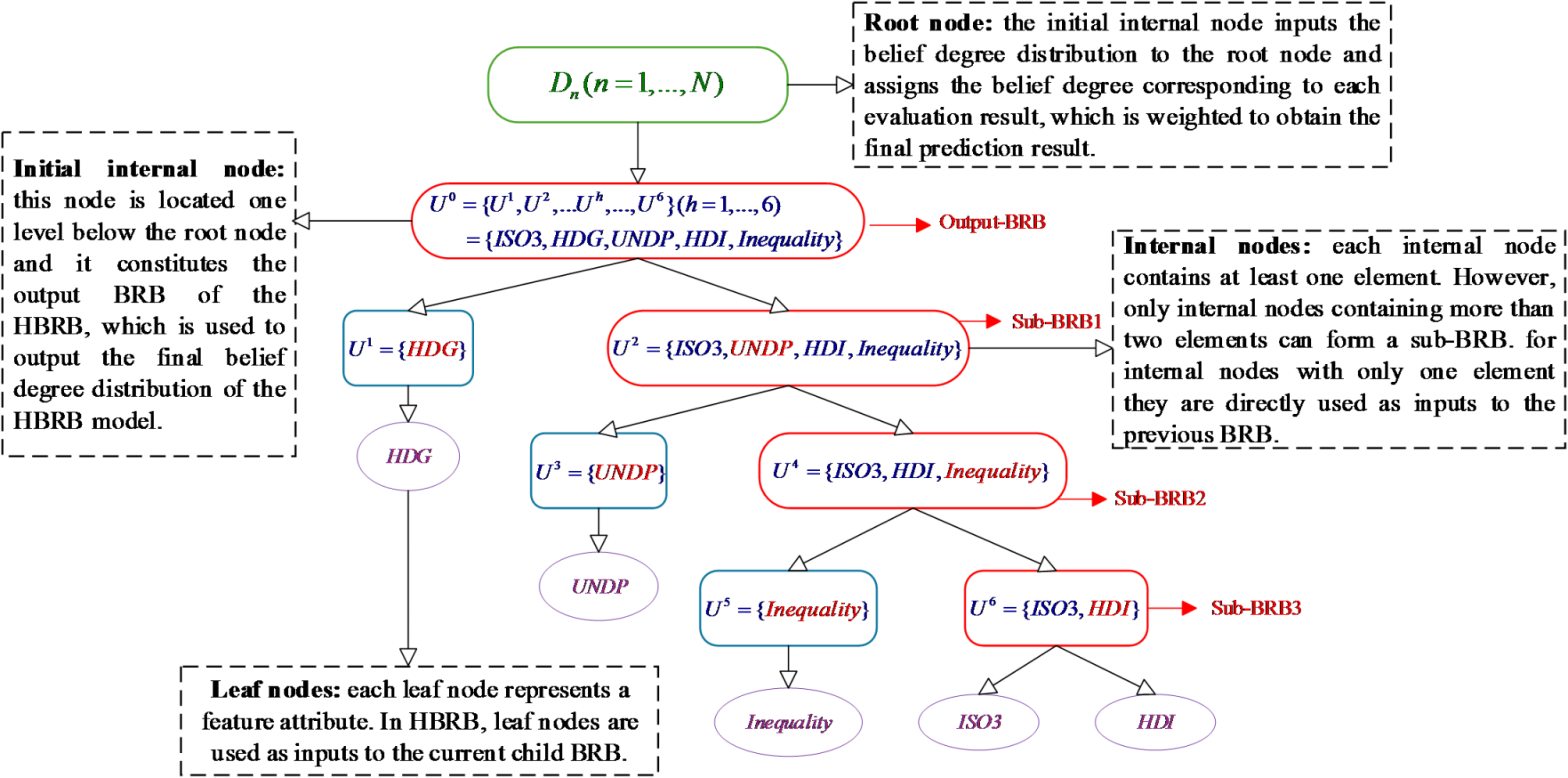
MTS Construction Process for Predicting Global Educational Inequality Components.

After the above three steps, the MTS structure has been constructed, to facilitate the reader’s understanding, the following is a case study of predicting global educational inequality and explains in detail how to construct the MTS-based HBRB model. Among them, the five features of country code (ISO3), human development groups (HDG), UNDP developing regions (UNDP), human development index (HDI), and global education inequality data (Inequality) integrated over the years are used as input attributes. The constructed MTS structure for this case is shown in Fig. 2.

1. Define the root node, leaf node and initial internal node: the root node is the set of all evaluation result levels, in this case, the evaluation results predicting global educational inequality can be categorized into {Low(L),Moderate(M),High(H)} three results. Therefore $D_{n}$ is denoted as $D_{n} = \{ L,M,H\}$. Leaf nodes are denoted as a collection of feature attributes, each individual leaf node represents a feature attribute, thus there are 5 leaf nodes in total. The initial internal node then contains all internal nodes, which are added to the initial internal node by subsequent generation.
2. Calculate the AR value and select the main element: step 3.2 is used to calculate the AR value between each feature as shown in Table 1. Assuming that the size of each internal node is selected in this case $\varepsilon$ = 2. Then firstly, the first two elements with the smallest sum of AR values are selected as the main elements according to Table 1, here HDG and UNDP are selected as the main elements to construct the internal nodes $U^{1}$ and $U^{2}$, and then the rest of the elements are added to the corresponding internal nodes according to their relevance to the main elements.
3. Optimizing internal nodes and generating leaf nodes: since the number of elements in $U^{2}$ exceeds the limit of $\varepsilon$, recalculate their AR values for the elements in $U^{2}$ and select the two main elements as the internal nodes of the next layer. And so on until the size of all internal nodes satisfies $\varepsilon$. Then each internal node is generated as a corresponding leaf node as shown in Fig. 2.
4. Construct sub-BRBs as well as output BRBs. in MTS, each internal node with number of elements greater than or equal to 2 is constructed as a sub-BRB, and the inputs of the sub-BRBs can be either the leaf nodes or the outputs of the sub-BRBs at the next level. Taking $U^{6}$ and $U^{4}$ as an example, the leaf nodes ISO3 and HDI serve as the inputs that constitute the sub-BRB in which $U^{6}$ is located, while the inputs of $U^{4}$ are composed of the leaf nodes Inequality and the outputs of $U^{6}$. By analogy, $U^{0}$ is used as the output BRB for the overall output of the model, and the final belief degree is calculated to be passed to the root node $D_{n}$.

**Table 1:** The AR of selected attributes of global educational inequality.

AR	ISO3	HDG	UNDP	HDI	Inequality	Sum (AR)
ISO3	1	0.27043	0.347842	0.952125	0.769613	3.34001
HDG	0.27043	1	0.192453	0.284028	0.220381	1.967292
UNDP	0.347842	0.192453	1	0.323844	0.280493	2.144633
HDI	0.952125	0.284028	0.323844	1	0.739929	3.299925
Inequality	0.769613	0.220381	0.280493	0.739929	1	3.010417

### The reasoning process of HBRB-I

After the MTS-based HBRB-I construction in Section “HBRB-I model construction based on MTS”, the inference process for each sub-BRB and output BRB is shown below:

*Step 1 (Transforming Input Information)*: In BRB, the input information needs to be transformed into a form that can be processed by the confidence rule and this information is processed during the inference process, which is generally done by converting the information obtained from sampling to affiliation via an affiliation function, described as follow:

7 $$\left\{ \begin{gathered} \alpha_{i,j} = (A_{i,j + 1} - x_{i} ) \cdot (A_{i,j + 1} - A_{i,j} )^{ - 1} \quad \;{\text{A}}_{i,j} \le x_{i} \le A_{i,j + 1} ,j = 1,2, \ldots ,J_{i} - 1 \hfill \\ \alpha_{i,j + 1} = 1 - \alpha_{i,j} \quad \quad \quad \quad \quad \quad \quad \quad \quad \quad \;{\text{A}}_{i,j} \le x_{i} \le A_{i,j + 1} ,j = 1,2, \ldots ,J_{i} - 1 \hfill \\ \alpha_{i,s} = 0\quad \quad \quad \quad \quad \quad \quad \quad \quad \quad \quad \quad \quad \quad s = 1,2, \ldots ,J_{i} ,s \ne j,j + 1 \hfill \\ \end{gathered} \right.$$

The final belief distribution is described as follows:

8 $$S(x_{i} ) = \{ (A_{i,j} ,\alpha_{i,j} ),i = 1, \ldots ,M;j = 1, \ldots ,J_{i} \}$$

where $A_{i,j}$ represents the *j*th reference value corresponding to the *i*th input, and $\alpha_{i,j}$ is the membership degree of the corresponding reference value.

*Step 2 (Rule activation weight)*: Based on the match, the activation weight of each rule is calculated. The activation weight reflects the effectiveness of each rule under the current input conditions. Typically, the activation weight is a function of the matching degree and the rule’s own belief degree:

9 $$\omega_{k} = \theta_{k} \left( {\prod\limits_{i = 1}^{M} {(\alpha_{i}^{k} )^{{\overline{{\delta_{i} }} }} } } \right) \cdot \left( {\sum\limits_{l = 1}^{L} {\theta_{l} \prod\limits_{i = 1}^{M} {(\alpha_{i}^{k} )^{{\overline{{\delta_{i} }} }} } } } \right)^{ - 1} ,\overline{{\delta_{i} }} = \frac{{\delta_{i} }}{{\mathop {\max }\limits_{i = 1, \ldots ,M} \{ \delta_{i} \} }}$$

where $\theta_{k} \in [0,1][0,1]$ is the rule weight of the *k*th rule, $\overline{{\delta_{i} }}$ is the normalized weight.

*Step 3 (Generate result belief degree)*: The belief degree $\hat{\beta }_{n} (n = 1, \ldots ,N)$ of the result is generated by the analytic ER algorithm fused with the activation rule.

10 $$\begin{gathered} \hat{\beta }_{n} = {{\left( {\eta \times \left[ {\prod\limits_{k = 1}^{L} {\left( {\omega_{k} \beta_{n,k} + 1 - \omega_{k} \sum\limits_{i = 1}^{N} {\beta_{i,k} } } \right)} - \prod\limits_{k = 1}^{L} {\left( {1 - \omega_{k} \sum\limits_{i = 1}^{N} {\beta_{i,k} } } \right)} } \right]} \right)} \mathord{\left/ {\vphantom {{\left( {\eta \times \left[ {\prod\limits_{k = 1}^{L} {\left( {\omega_{k} \beta_{n,k} + 1 - \omega_{k} \sum\limits_{i = 1}^{N} {\beta_{i,k} } } \right)} - \prod\limits_{k = 1}^{L} {\left( {1 - \omega_{k} \sum\limits_{i = 1}^{N} {\beta_{i,k} } } \right)} } \right]} \right)} {\left( {1 - \eta \times \left[ {\prod\limits_{k = 1}^{L} {(1 - \omega_{k} )} } \right]} \right)}}} \right. \kern-0pt} {\left( {1 - \eta \times \left[ {\prod\limits_{k = 1}^{L} {(1 - \omega_{k} )} } \right]} \right)}} \hfill \\ \eta = \left[ {\sum\limits_{j = 1}^{N} {\prod\limits_{k = 1}^{L} {\left( {\omega_{k} \beta_{j,k} + 1 - \omega_{k} \sum\limits_{i = 1}^{N} {\beta_{i,k} } } \right)} } - (N - 1)\prod\limits_{k = 1}^{L} {\left( {1 - \omega_{k} \sum\limits_{i = 1}^{N} {\beta_{i,k} } } \right)} } \right]^{ - 1} \hfill \\ \end{gathered}$$

*Step 4*: The final output distribution is generated based on the belief degree of the evaluation results:

11 $$S(x) = \{ (D_{n} ,\hat{\beta }_{n} ),n = 1, \ldots ,N\}$$

The expected utility of $S(x)$ in Eq. (12) is determined by the utility of a single evaluation result $D_{n}$, denoted as $\mu (D_{n} )$:

12 $$\mu (S(x)) = \sum\limits_{n = 1}^{N} {\mu (D_{n} )\beta_{n} }$$

### Interpretable definition of HBRB-I

In artificial intelligence, model interpretability is classified into ante-hoc and post-hoc interpretability. Ante-hoc interpretability refers to the transparency and understandability of the model’s structure and inference process, achieved through design and parameter settings during the model’s construction. It stresses that the model should have clear logic and intuitive rules, allowing experts and users to understand how conclusions are drawn from the input data^29,30^.

In the HBRB-I model, ante-hoc interpretability primarily involves reference value differentiation, rule completeness, a transparent inference engine, and rule base simplicity. The HBRB-I model uses a transparent ER inference engine, and its hierarchical structure simplifies the rule base. Rule completeness ensures that all possible input scenarios are covered, with at least one rule activated, meaning that the model must account for all possible system states. Distinctness of reference values ensures that these values and fuzzy membership functions define distinct domains within the BRB model, allowing clear interpretation of the state of each input variable. In practice, models often encounter complex input variables with multiple potential states. For accurate simulation and prediction, the model must distinguish between these states. For example, in a diagnostic system, variables such as symptom severity, patient age, and body temperature must be clearly identified to accurately assess conditions. Typically, BRB models are set by domain experts, but these initial values may not be optimal. Given the complexity and variety of real-world data, reference values may need to be adjusted to better reflect actual conditions. In addition, because the reference values influence how the model translates inputs into relationships, an adaptive optimization strategy is essential to improve model performance and decision confidence.

13 $$\begin{gathered} A_{i,j - 1} \le lb(A_{i,j} ) \le A_{i,j} \le ub(A_{i,j} ) \le A_{i,j + 1} \hfill \\ or\;\;A_{i,j - 1} \ge ub(A_{i,j} ) \ge A_{i,j} \ge lb(A_{i,j} ) \ge A_{i,j + 1} \hfill \\ \end{gathered}$$

Equation (13) gives the interpretable optimization constraint strategy for reference values, which should be strictly monotonic for each attribute, and for reference value $A_{i,j}$ it should have an optimization interval, where $ub(A_{i,j} )$ denotes the upper bound and $lb(A_{i,j} )$ denotes the lower bound, where the upper bound should not be higher than the next reference value and the upper bound should not be lower than the previous reference value.

Post-hoc interpretability refers to the model’s ability to produce transparent and interpretable outputs during the application and analysis phases, helping users to understand and validate the model’s decisions. It emphasizes building user confidence by making the outputs of the model interpretable after execution^31^. In the case of HBRB-I, post-hoc interpretability involves ensuring that the structure and parameters of the model have physical meaning and that its rules exhibit consistency. The structure of the HBRB-I model must maintain logical consistency, allowing for the distinction between different levels of rules-where high-level rules handle global decisions and low-level rules address specific details. In terms of parameters, these include attribute weights, rule weights, and rule belief degrees, which are typically based on expert knowledge. However, these parameters must also be adapted to the actual conditions of the system. The interpretability of these parameters is crucial for the model to maintain meaningful and transparent decision making. Specifically, the limits of parameter interpretability are expressed as follows:

14 $$\begin{gathered} 0 \le \theta_{k} ,\delta_{i} ,\beta_{n,k} \le 1 \hfill \\ \beta_{n,k} \in \{ \{ \beta_{1,k} \le \beta_{2,k} \le \cdots \le \beta_{N,k} \} or\{ \beta_{1,k} \le \cdots \le \max (\beta_{1,k} ,\beta_{2,k} , \ldots ) \ge \cdots \ge \beta_{N,k} \} \;or \hfill \\ \quad \quad \quad \{ \beta_{1,k} \ge \beta_{2,k} \ge \cdots \ge \beta_{N,k} \} \} \hfill \\ \end{gathered}$$

where the rule weight $\theta_{k}$, attribute weight $\delta_{i}$, and rule belief degree $\beta_{n,k}$ should be between 0 and 1, and for $\beta_{n,k}$ should satisfy nonconcavity, i.e., take the form of a monotonic or convex function. The reason is that the two endpoints in a confidence rule often indicate two contradictory belief degrees, so there cannot be a situation where two contradictory belief degrees are high at the same time.

Furthermore, the ante-hoc interpretability of the HBRB-I model essentially stems from the high degree of alignment of its rule system with expert domain knowledge. However, this property also results in the explanatory validity of the model being directly dependent on the accuracy and reliability of expert knowledge. If there is bias or uncertainty in expert knowledge (e.g., miscalculation of the drivers of educational inequality), the rules generated by the model may deviate from the true causal mechanisms, which in turn erodes the decision maker’s trust in the model’s output.

To address this issue, this study proposes a multi-expert knowledge consensus mechanism. It balances single-individual cognitive differences by weighted aggregation of multi-expert knowledge, ensuring greater interpretability of the model and improving the reliability of predictions. Specifically, an reliability factor of the experts is introduced $\kappa_{{{\text{expert}}}}$. It can be quantified by the following:

$$0 \le \kappa_{{{\text{expert}}}} \le 1$$

where $\kappa_{{{\text{expert}}}}$ denotes the expert knowledge reliability, which is a statistical concept and should be adjusted in long-term testing and practice. $\kappa_{{{\text{expert}}}}$ is closer to 1, indicating that the expert knowledge is more accurate and reliable, which suggests that real systems can be accurately modeled with expert knowledge. On the contrary, it indicates that the expert knowledge is not reliable enough, then the BRB with optimization process degrades to a data-driven model.

Under the guidance of the multi-expert consensus mechanism, the initial parameters of HBRB-I are weighted and aggregated from multiple expert knowledge. Taking the belief degree $\beta_{n,k}$ of a rule as an example, suppose three experts give their respective expert knowledge $\beta_{n,k}^{1} ,\beta_{n,k}^{2} ,\beta_{n,k}^{3}$, and the reliability factor of the three experts’ knowledge is $\kappa_{{{\text{expert}}}}^{1} ,\kappa_{{{\text{expert}}}}^{2} ,\kappa_{{{\text{expert}}}}^{3}$. Then the belief degree after the aggregation by the multi-expert consensus mechanism is:

15 $$\beta_{n,k} = \frac{{\sum\nolimits_{e = 1}^{E} {\kappa_{{{\text{expert}}}}^{e} \beta_{n,k}^{e} } }}{{\sum\nolimits_{e = 1}^{E} {\kappa_{{{\text{expert}}}}^{e} } }}$$

where $e(1, \ldots ,E)$ denotes the expert knowledge given by the eth expert. For the other parameters of the HBRB-I model can also be obtained in this way. Through this weighted aggregation method, multiple expert knowledge can be effectively integrated so that the model can balance multiple expert opinions and correct unreliable cognitive biases.

## Interpretable optimization algorithm construction

In this study, the Projected Covariance Matrix Adaptive Evolutionary Strategy (P-CMA-ES) is used as the global optimization algorithm^32,33^. This algorithm’s improved covariance matrix adaptation mechanism enhances both robustness and efficiency, making it particularly effective for high-dimensional and complex optimization problems. In this paper, the Mean Squared Error (MSE) is selected as the global optimization objective, described as follow:

16 $$\min \, \xi_{MSE} (\Omega ) = \frac{1}{T}\sum\limits_{t = 1}^{T} {(y - \hat{y})^{2} } ,\Omega = \{ \beta ,\theta ,w,A\}$$

where *y* is the predicted value of the model output, $\hat{y}$ is the true value of the system, and $\Omega$ is the set of parameters to be optimized.

The specific procedure of the P-CMA-ES optimization algorithm is shown below:

*Step 1 (Initialize the optimization target parameters)*: The set of target parameters can be expressed as:

17 $$\Omega^{0} = \{ A_{1,1} , \ldots ,A_{{M,J_{M} }} ,\beta_{1,1} , \ldots ,\beta_{N,L} , \ldots ,\theta_{1} , \ldots ,\theta_{L} , \ldots ,w_{1} , \ldots ,w_{M} \}$$

*Step 2 (Sampling)*: Sampling to obtain data for each generation:

18 $$\Omega_{k}^{(g + 1)} = mean^{(g)} + \varepsilon^{(g)} N(0,C^{(g)} ),k = 1,2, \ldots ,\lambda$$

where $\Omega_{k}^{(g + 1)}$ is the *k*th solution in the (*g* + 1) generation, *N* denotes the normal distribution, $C^{(g)}$ denotes the *g*th generation covariance matrix, $\varepsilon^{(g)}$ denotes the step size of the gth generation, and $mean^{(g)}$ denotes the mean of the *g*th generation, and since the solutions are randomly generated.

*Step 3 (interpretability constraints)*: According to the interpretability analysis in Section “Modeling process of HBRB-I based on multilayer tree structure”, the parameters of the BRB are often provided by expert knowledge. However, the stochastic nature of the optimization algorithm can lead to model parameters that appear to contradict this expert knowledge. Therefore, constraints need to be added to ensure the interpretability of the model.

19 $$\begin{gathered} \sum\limits_{n = 1}^{N} {\beta_{n,k} } = 1;0 \le \beta_{n,k} ,\theta_{k} ,w_{i} \le 1, \hfill \\ \beta_{n,k} \in \{ \{ \beta_{1,k} \le \beta_{2,k} \le \cdots \le \beta_{N,k} \} or\{ \beta_{1,k} \ge \beta_{2,k} \ge \cdots \ge \beta_{N,k} \} \} \hfill \\ or\quad \quad \{ \beta_{1,k} \le \cdots \le \max (\beta_{1,k} ,\beta_{2,k} , \ldots ) \ge \cdots \ge \beta_{N,k} \} \hfill \\ \end{gathered}$$

where $\varpi_{n,k}$ denotes the belief degree of the nth result in the kth rule under constraints.

*Step 4 (Projection operation)*: To satisfy the equational constraints, use the projection operation to transform the equational constraints into equational constraints in the hyperplane:

20 $$\begin{gathered} A_{e} \Omega_{k}^{(g + 1)} (1 + n_{e} \times (j - 1):n_{e} \times j) = 1,j = 1,2, \ldots ,N + 1 \hfill \\ \Omega_{k}^{(g + 1)} (1 + n_{e} \times (j - 1):n_{e} \times j) = \Omega_{k}^{(g + 1)} (1 + n_{e} \times (j - 1):n_{e} \times j) \hfill \\ - A_{e}^{T} \times (A_{e} \times A_{e}^{T} )^{ - 1} \times \Omega_{k}^{(g + 1)} (1 + n_{e} \times (j - 1):n_{e} \times j) \times A_{e} \hfill \\ \end{gathered}$$

*Step 5 (Select Optimal Solution)*

21 $$mean^{(g + 1)} = \sum\limits_{i = 1}^{\mu } {\omega_{i} \Omega_{i:\lambda }^{(g + 1)} }$$

*Step 6 (Update the covariance matrix)*

22 $$\begin{gathered} C^{(g + 1)} = (1 - c_{1} - c_{2} ) \cdot C^{(g)} + c_{1} p_{c}^{(g + 1)} (p_{c}^{(g + 1)} )^{T} + c_{2} \sum\limits_{i = 1}^{\tau } {\omega_{i} \left( {\frac{{\Omega_{i:\lambda }^{(g + 1)} - mean^{(g)} }}{{\varepsilon^{(g)} }}} \right)} \left( {\frac{{\Omega_{i:\lambda }^{(g + 1)} - mean^{(g)} }}{{\varepsilon^{(g)} }}} \right)^{T} \hfill \\ p_{c}^{(g + 1)} = (1 - c_{c} ) \cdot p_{c}^{(g)} + \sqrt {c_{c} (2 - c_{c} )} \cdot \left( {\sum\limits_{i = 1}^{\tau } {\omega_{i}^{2} } } \right)^{{ - \frac{1}{2}}} \cdot \, \frac{{\left( {mean^{(g + 1)} - mean^{(g)} } \right)}}{{\varepsilon^{(g)} }} \hfill \\ \end{gathered}$$

Finally, the above six-step process is recursively executed until the optimization is complete.

## Case study

The background of the dataset is given in Section “Background on the dataset”, the construction of the initial HBRB-I model is given in Section “Initial HBRB-I model construction”, the performance analysis of the model is given in Section “Performance analysis of the model”, and the interpretability analysis of the model is given in Section “Supplementary cases”.

### Background on the dataset

In today’s globalized world, educational inequality highlights the persistent disparities between nations and communities. Despite progress in expanding access to education, many children, particularly in low-income and conflict-affected areas, still lack opportunities to learn. The quality of education also varies widely, with well-resourced schools in affluent areas and under-resourced schools in marginalized regions. Gender inequality exacerbates the situation, as societal norms and economic challenges often limit girls’ access to education in certain cultures. Addressing education inequality is not only a matter of fairness, but is essential to fostering equitable societies and empowering individuals to contribute to personal and national growth. This study selects the United Nations Human Development Report dataset (2010–2021) for its unique alignment with the requirements of the HBRB-I model, which prioritizes interpretable rule extraction and dynamic multi-scale analysis. The dataset’s design addresses three critical needs for our methodology: (1) Causal Traceability: The inclusion of both Human Development Index (HDI) rankings and historical educational inequality indices (2010–2021) allows the model to establish rules that explicitly link socio-economic drivers (e.g., HDI < 0.55) to inequality outcomes. (2) Policy Integration Readiness: The dataset’s direct mapping to UN Sustainable Development Goal (SDG) monitoring frameworks (e.g., SDG 4.5 on equity) allows HBRB-I outputs to bypass complex translation steps. (3) Compared to crowdsourced alternatives, this dataset’s expert-curated structure—including conflict-adjusted HDI values and UN-validated inequality metrics—minimizes noise from inconsistent data collection practices, a key requirement for generating reliable rules. Licensed under the Creative Commons Attribution 3.0 IGO License, it further ensures compliance with open science standards while protecting sensitive geopolitical classifications^34^.

### Initial HBRB-I model construction

In this dataset, data on educational inequality in 197 countries around the world are recorded, and to better predict future educational inequality, we selected educational inequality (IE) from 2013 to 2020 as well as Human Development Index (HDI) rankings in 2021 as input attributes to predict educational inequality in 2021 for each country and region.

**Fig. 3:**
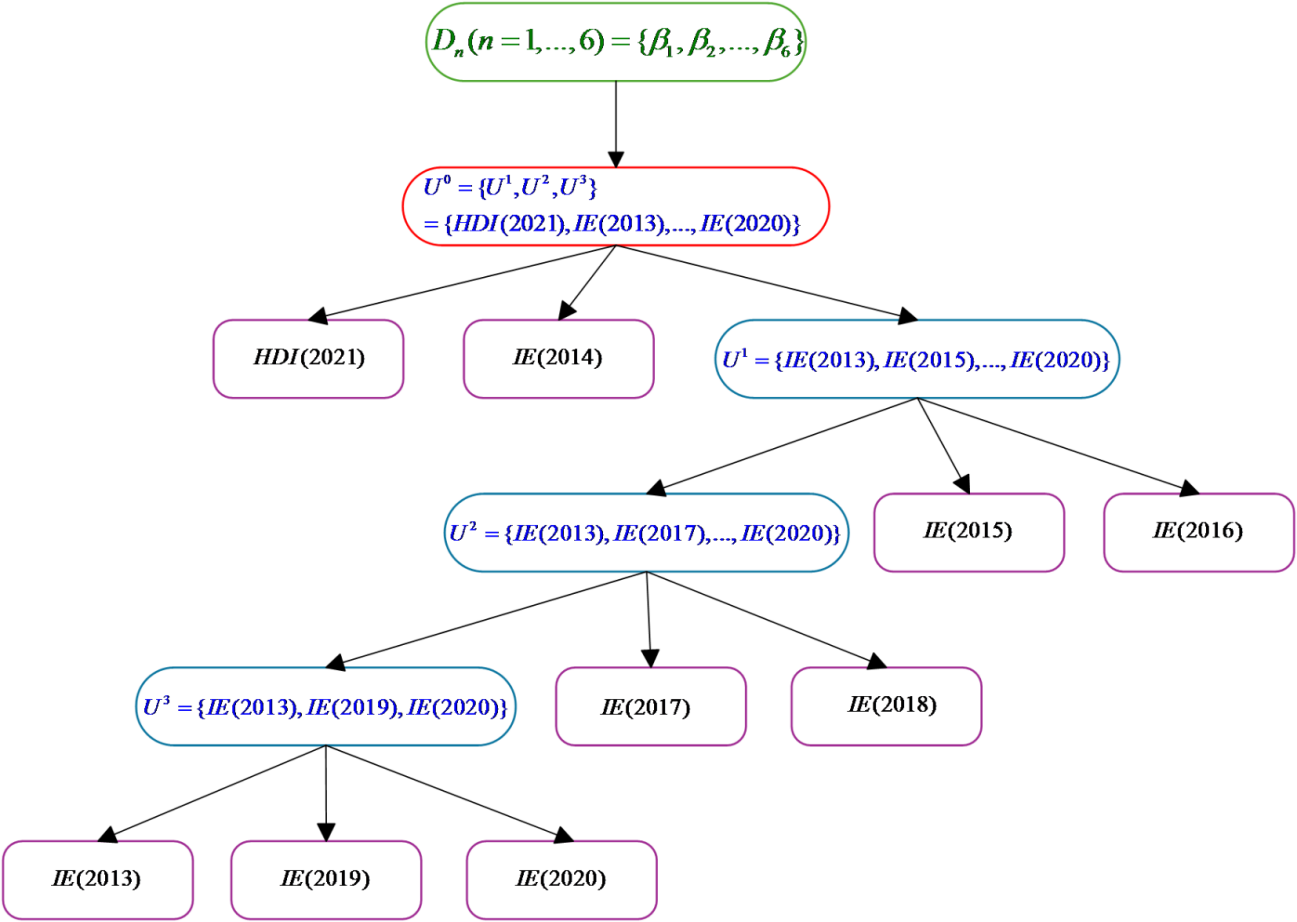
Schematic of the MTS structure for predicting educational inequality.

The MTS structure proposed in Section “HBRB-I model construction based on MTS” is utilized to construct the HBRB-I model, and the constructed MTS structure diagram is shown in Fig. 3. In the construction of the MTS, the size of the internal node $\varepsilon$ is set to 3. The root node is defined as $\{ \beta_{1} ,\beta_{2} , \ldots ,\beta_{6} \}$ according to the steps given in Section “HBRB-I model construction based on MTS”. The leaf node is defined as $\{ HDI(2021),IE(2013), \ldots ,IE(2021)\}$. Based on the calculated AR values, HDI(2021), IE(2013) and IE(2014) are first selected as the main elements, and then the remaining elements are added to the internal node where the main elements are located in sequence according to the size of the AR values in relation to the three main elements. As can be seen from the figure, the remaining elements are added to the internal node where IE(2013) is located, at which point the size of the node exceeds $\varepsilon$. Therefore, the node needs to be processed to generate the next level of internal nodes in $U^{1}$ according to the computation given in Section “HBRB-I model construction based on MTS”, and so on, until the size of all the internal nodes no longer exceeds s. It is important to note that the figure is different from the previous presentation of the MTS is that internal nodes with only one element become leaf nodes directly; this is done to simplify the information in the figure, which does not affect the model itself.

**Fig. 4:**
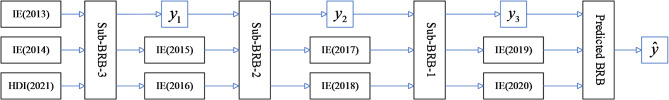
Modeling process of the HBRB-I model for educational inequality prediction.

The HBRB-I model constructed based on the MTS structure is shown in Fig. 4.Compared with the traditional BRB structure, the HBRB-I model adopts a hierarchical structure, which can greatly reduce the size of the rule base, as can be seen in Fig. 4, using the structure of three layers of sub-BRBs and a predictive BRB model, in which each BRB contains $3^{3} = 27$ rules, which generates a total of 108 rules, while if we adopt the traditional BRB modeling approach, the number of rules is determined by the Cartesian product of the reference values, then the number of rules will generate 108 rules, which obviously reduces the number of rules. $3^{9} = 19683$ rules would be generated, which would obviously reduce the processing speed of the model significantly.

In terms of parameter definition, the reference value of each input feature corresponds to the trend change of the feature, in the HBRB-I model, three reference points are defined for each feature, which are {Low, Medium, High}, and the specific distribution of the reference value is shown in Table 2, while the reference value corresponding to the result is assigned to six reference points, for evaluation levels can be categorized as {very equal(VE), equal(E), low inequality(LIE), medium inequality(MIE), higher inequality(HIE), very high inequality(VHIE)}, and the specific distribution is shown in Table 3. The rule base of HBRB-I combines the three domains aggregated through the multi-expert consensus mechanism in Section “Interpretable definition of HBRB-I”, in this case, the reliability of the three experts’ knowledge is 0.9, 0.8 and 0.9. Take the predicted BRB in HBRB-I as an example, where the rule base of the first two expert knowledge is given in Table 4, the rule base of the third expert knowledge is given in Table 5 as well as the rule base of the HBRB-I model obtained through the aggregation of the multi-expert knowledge consensus mechanism.

**Table 2:** Distribution of reference values for input attributes.

Attribute	Low	Medium	High
IE(2013–2020)	1.37	25.07	50.15
HDI(2021)	0	0.5	1

**Table 3:** Reference value distribution of results.

Result attributes	$\beta_{1}$ (VE)	$\beta_{2}$ (E)	$\beta_{3}$ (LIE)	$\beta_{4}$ (MIE)	$\beta_{5}$ (HIE)	$\beta_{6}$ (VHIE)
Resulting reference value	1	10	20	30	40	50.2

**Table 4:** Rule base of knowledge for the first two experts.

No.	First expert knowledge rule base							Second expert knowledge rule base						
	$\theta_{k}$	$\{ \beta_{1} ,\beta_{2} ,\beta_{3} ,\beta_{4} ,\beta_{5} ,\beta_{6} \}$						$\theta_{k}$	$\{ \beta_{1} ,\beta_{2} ,\beta_{3} ,\beta_{4} ,\beta_{5} ,\beta_{6} \}$					
1	0.7	0.12	0.23	0.30	0.20	0.11	0.05	0.7	0.05	0.12	0.41	0.27	0.14	0.01
2	1.0	0.86	0.14	0.00	0.00	0.00	0.00	1.0	0.74	0.18	0.08	0.00	0.00	0.00
3	0.7	0.07	0.11	0.25	0.49	0.05	0.03	0.8	0.05	0.15	0.22	0.39	0.19	0.00
4	0.7	0.12	0.19	0.35	0.16	0.12	0.06	0.9	0.07	0.16	0.25	0.32	0.19	0.02
5	0.8	0.09	0.38	0.40	0.08	0.03	0.02	0.8	0.08	0.32	0.21	0.20	0.11	0.08
6	0.7	0.03	0.14	0.39	0.27	0.17	0.00	0.8	0.02	0.31	0.24	0.19	0.15	0.09
7	0.8	0.06	0.16	0.30	0.21	0.19	0.08	0.8	0.07	0.31	0.32	0.18	0.12	0.00
8	0.7	0.14	0.16	0.17	0.33	0.14	0.07	0.7	0.04	0.09	0.30	0.33	0.17	0.08
9	0.9	0.01	0.14	0.29	0.40	0.10	0.06	0.8	0.03	0.15	0.50	0.19	0.13	0.00
10	0.7	0.00	0.14	0.16	0.32	0.30	0.07	0.8	0.11	0.12	0.38	0.22	0.11	0.05
11	0.6	0.02	0.11	0.28	0.47	0.12	0.00	0.7	0.11	0.12	0.26	0.26	0.24	0.00
12	0.9	0.04	0.25	0.37	0.23	0.07	0.04	0.7	0.05	0.12	0.18	0.27	0.32	0.07
13	0.7	0.08	0.32	0.28	0.16	0.11	0.06	0.9	0.01	0.17	0.34	0.33	0.11	0.05
14	0.6	0.13	0.26	0.25	0.20	0.13	0.03	0.7	0.11	0.14	0.25	0.22	0.18	0.11
15	0.7	0.19	0.26	0.25	0.22	0.08	0.00	0.7	0.01	0.17	0.20	0.43	0.19	0.00
16	0.7	0.01	0.05	0.30	0.50	0.13	0.01	0.7	0.00	0.11	0.32	0.29	0.21	0.08
17	0.7	0.00	0.05	0.08	0.29	0.41	0.17	0.6	0.00	0.08	0.31	0.23	0.21	0.16
18	0.7	0.00	0.29	0.27	0.22	0.20	0.02	0.9	0.09	0.11	0.27	0.24	0.16	0.13
19	1.0	0.05	0.06	0.20	0.46	0.22	0.00	0.9	0.08	0.30	0.25	0.22	0.15	0.00
20	0.8	0.04	0.05	0.21	0.34	0.25	0.10	0.6	0.14	0.22	0.39	0.16	0.08	0.02
21	0.8	0.04	0.25	0.30	0.25	0.14	0.02	0.9	0.00	0.17	0.22	0.35	0.21	0.05
22	0.9	0.06	0.13	0.28	0.28	0.21	0.05	0.7	0.03	0.03	0.31	0.40	0.14	0.09
23	0.7	0.10	0.11	0.14	0.41	0.19	0.05	0.9	0.02	0.09	0.11	0.15	0.28	0.35
24	0.8	0.03	0.04	0.29	0.36	0.23	0.04	0.7	0.01	0.17	0.21	0.27	0.27	0.06
25	0.8	0.08	0.11	0.21	0.22	0.33	0.05	0.9	0.02	0.24	0.33	0.24	0.16	0.01
26	1.0	0.01	0.01	0.05	0.07	0.22	0.65	1.0	0.00	0.00	0.00	0.08	0.30	0.62
27	0.8	0.06	0.08	0.26	0.35	0.26	0.00	0.9	0.05	0.15	0.29	0.38	0.13	0.00

**Table 5:** The third expert knowledge rule base as well as the rule base for HBRB-I.

No.	Third expert knowledge rule base							HBRB-I rule base						
	$\theta_{k}$	$\{ \beta_{1} ,\beta_{2} ,\beta_{3} ,\beta_{4} ,\beta_{5} ,\beta_{6} \}$						$\theta_{k}$	$\{ \beta_{1} ,\beta_{2} ,\beta_{3} ,\beta_{4} ,\beta_{5} ,\beta_{6} \}$					
1	0.8	0.12	0.17	0.42	0.16	0.14	0.00	0.7	0.05	0.12	0.41	0.27	0.14	0.01
2	1.0	0.85	0.10	0.02	0.02	0.00	0.00	1.0	0.74	0.18	0.08	0.00	0.00	0.00
3	0.8	0.06	0.12	0.27	0.35	0.16	0.05	0.8	0.05	0.15	0.22	0.39	0.19	0.00
4	0.6	0.10	0.26	0.39	0.24	0.01	0.00	0.9	0.07	0.16	0.25	0.32	0.19	0.02
5	0.9	0.11	0.34	0.21	0.18	0.15	0.01	0.8	0.08	0.32	0.21	0.20	0.11	0.08
6	0.8	0.05	0.16	0.17	0.35	0.27	0.00	0.8	0.02	0.31	0.24	0.19	0.15	0.09
7	0.9	0.04	0.07	0.11	0.21	0.36	0.20	0.8	0.07	0.31	0.32	0.18	0.12	0.00
8	0.9	0.00	0.09	0.31	0.36	0.16	0.08	0.7	0.04	0.09	0.30	0.33	0.17	0.08
9	0.7	0.07	0.30	0.29	0.20	0.14	0.00	0.8	0.03	0.15	0.50	0.19	0.13	0.00
10	0.8	0.00	0.07	0.31	0.24	0.23	0.14	0.8	0.11	0.12	0.38	0.22	0.11	0.05
11	0.7	0.04	0.39	0.30	0.12	0.12	0.03	0.7	0.11	0.12	0.26	0.26	0.24	0.00
12	0.8	0.01	0.14	0.26	0.31	0.16	0.13	0.7	0.05	0.12	0.18	0.27	0.32	0.07
13	0.7	0.07	0.13	0.32	0.29	0.15	0.03	0.9	0.01	0.17	0.34	0.33	0.11	0.05
14	0.6	0.09	0.14	0.27	0.31	0.17	0.02	0.7	0.11	0.14	0.25	0.22	0.18	0.11
15	0.8	0.15	0.16	0.43	0.16	0.05	0.05	0.7	0.01	0.17	0.20	0.43	0.19	0.00
16	0.8	0.02	0.03	0.28	0.40	0.24	0.02	0.7	0.00	0.11	0.32	0.29	0.21	0.08
17	0.8	0.01	0.10	0.26	0.35	0.25	0.03	0.6	0.00	0.08	0.31	0.23	0.21	0.16
18	0.9	0.06	0.11	0.33	0.35	0.13	0.01	0.9	0.09	0.11	0.27	0.24	0.16	0.13
19	0.6	0.10	0.18	0.33	0.17	0.13	0.09	0.9	0.08	0.30	0.25	0.22	0.15	0.00
20	0.7	0.02	0.24	0.25	0.22	0.17	0.10	0.6	0.14	0.22	0.39	0.16	0.08	0.02
21	1.0	0.08	0.20	0.20	0.26	0.14	0.12	0.9	0.00	0.17	0.22	0.35	0.21	0.05
22	0.8	0.11	0.24	0.39	0.17	0.08	0.01	0.7	0.03	0.03	0.31	0.40	0.14	0.09
23	0.7	0.04	0.19	0.25	0.28	0.22	0.02	0.9	0.02	0.09	0.11	0.15	0.28	0.35
24	0.8	0.09	0.12	0.29	0.24	0.18	0.08	0.7	0.01	0.17	0.21	0.27	0.27	0.06
25	0.7	0.06	0.07	0.18	0.33	0.29	0.06	0.9	0.02	0.24	0.33	0.24	0.16	0.01
26	1.0	0.00	0.00	0.07	0.12	0.16	0.66	1.0	0.00	0.00	0.00	0.08	0.30	0.62
27	0.7	0.02	0.10	0.41	0.27	0.11	0.10	0.9	0.05	0.15	0.29	0.38	0.13	0.00

### Performance analysis of the model

**Fig. 5:**
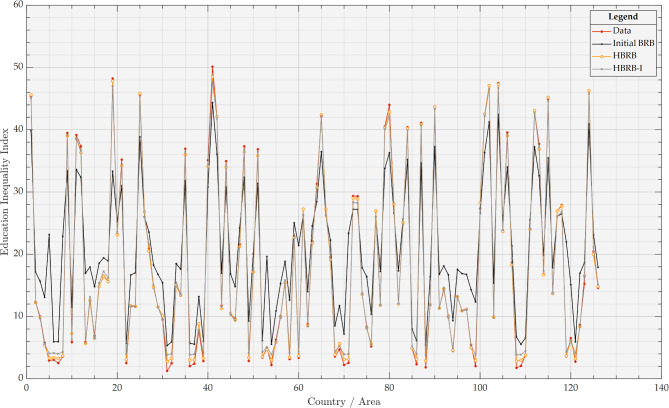
Analysis of prediction plots for each BRB model.

After constructing the HBRB-I model, it was trained using an interpretable optimization algorithm. To evaluate its performance, the HBRB-I model, the HBRB model using the standard optimization algorithm, and the original BRB model were compared. The dataset was cross-validated with a 50% discount and predictions were made for 126 countries and regions. The performance of the models is shown in Table 6 and the visual analysis is shown in Fig. 5. As shown in Table 6 and Fig. 5, the accuracy performance of the initial BRB model based on expert knowledge is 92.87%. This indicates that the expert knowledge obtained from the aggregation based on the multi-expert consensus mechanism is reliable and effective. The two models of HBRB-I and HBRB adopt the interpretable optimization algorithm and the common optimization algorithm that only aims at accuracy, respectively, but the performance difference between the two after optimization is not much, and both of them show good performance, which indicates that the interpretable optimization algorithm proposed in this paper will not cause too much loss of model performance due to the maintenance of the interpretability. Interpretability leads to too much performance loss of the model.

**Table 6:** Comparison of the performance of the models.

Model	MSE	MAE	*R*^2^
Initial BRB	15.5638 (± $0.9917$)	3.4819 (± $0.1383$)	0.9287 (± $0.0164$)
HBRB	0.4830 (± $0.3698$)	0.4693 (± $0.1673$)	0.998 (± $0.0012$)
HBRB-I	0.5322 (± $0.3668$)	0.5366 (± $0.1423$)	0.9978 (± $0.0011$)
RF	0.6858 (± $0.4188$)	0.5478 (± $0.1722$)	0.9967 (± $0.0016$)
SVM	0.1587 (± $0.0645$)	0.2239 (± $0.0397$)	0.9991 (± $0.0005$)
KNN	2.1252 (± $0.8652$)	0.9843 (± $0.1450$)	0.9896 (± $0.0047$)
BPNN	24.3156 (± $6.9857$)	4.1298 (± $0.5324$)	0.8705 (± $0.0569$)

**Fig. 6:**
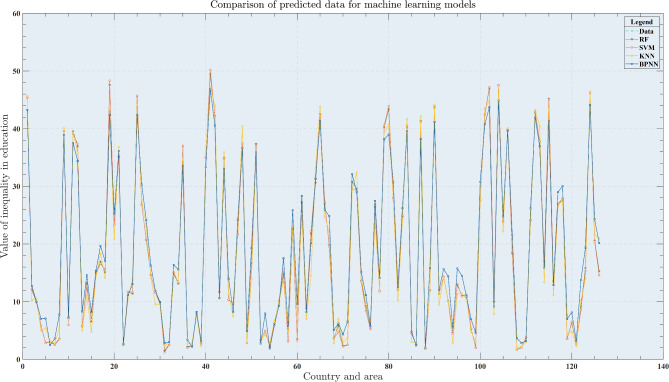
Predictive graph analysis for each machine learning model.

To further validate the HBRB-I model and assess the reliability of its interpretable optimization algorithms, we compare the BRB model with several mainstream machine learning models, including random forest (RF), support vector machine (SVM), k-nearest neighbor classification (KNN), and backpropagation neural network (BPNN). The performance of these machine learning models is shown in Table 6 and their predicted values are shown in Fig. 6. Due to the different principles and optimization techniques used by different models, some models (e.g., BPNN) tend to perform better on linear data, leading to significant differences in the prediction results. The KNN, SVM, and RF models show superior prediction performance, but are considered black-box models that lack interpretability. On the other hand, the HBRB-I model combines high accuracy and interpretable optimization algorithms that are both transparent and effective. This comparative analysis further validates the effectiveness of the HBRB-I model, highlighting its balance between accuracy and interpretability.

To evaluate the robustness of the models, 10 optimization iterations were performed and the results are summarized in Table 7. The two BRB models exhibit different performance characteristics. The HBRB model exhibits greater volatility due to the lack of constraints in its optimization, which leads to a larger search space, increasing the chance of finding multiple solutions but weakening robustness. In contrast, the improved HBRB-I model shows better stability with less fluctuation because the interpretable optimization algorithm adds constraints, which improves robustness. Compared to the HBRB model, machine learning models show more performance fluctuations during optimization, especially over multiple iterations. This may be partly due to randomness in training, such as random weight initialization. However, some models, such as those using deterministic gradient descent, can produce more consistent results. SVM models are more stable due to their integration strategy. While machine learning models can perform better in certain cases due to their flexibility, this same flexibility can lead to inconsistent results across different datasets, compromising robustness.

**Table 7:** 10 optimization analyses for each model.

Model	MSE			MAE			*R*^2^		
	Min	Average	Max	Min	Average	Max	Min	Average	Max
HBRB	0.5340	0.9299	2.123	0.5655	0.7826	1.2423	0.9880	0.9947	0.9969
HBRB-I	0.1988	0.3511	0.6594	0.3643	0.5948	0.67	0.9962	0.9980	0.9988
RF	0.3241	0.6858	1.4607	0.3732	0.5478	0.8156	0.9943	0.9967	0.9988
SVM	0.0479	0.1587	0.2472	0.1459	0.2239	0.2569	0.9984	0.9991	0.9998
KNN	1.0224	2.1252	3.4297	0.7964	0.9843	1.1582	0.9805	0.9896	0.9933
BPNN	16.6960	24.3156	35.2119	3.4653	4.1298	4.9331	0.8002	0.8705	0.9395

### Supplementary cases

To comprehensively evaluate the generalization ability of the HBRB-I model, four datasets from different domains in the UCI Machine Learning Database were selected as complementary cases in this study. The specific datasets are shown in Table 8.

**Table 8:** UCI dataset information.

Dataset	Number of antecedent attribute	Number of classified results	Industry applications	Sample size
Contraceptive	9	3	Medical classification forecast	1473
Ecoli	7	2	Biological sciences	336
Glass	9	6	Glass Identification	214
Seeds	7	3	Agricultural forecasts	210

In the experiments, a five-fold cross-validation method is used to evaluate the performance of the HBRB-I model, and the mean score accuracy is used as the final experimental result. Meanwhile, to better reflect the effectiveness of the HBRB-I model, we conducted a comparative analysis with other BRB models, including the extended belief rule-based system proposed by Liu et al.^35^, two dynamic rule activation DRA-EBRB systems proposed by Calzada et al.^36^ and greedy rule generation and activation method (GSR-BRB)^37^. The reference values for the antecedent attributes of all BRB models are set to three, while the reference values for the posterior attributes are divided according to the number of categorization results in different datasets. Two machine learning models, decision tree and random forest, are also added for comparative analysis. This section uses classification accuracy (Accuracy) and the number of rules as evaluation objectives. In this case, the performance of the model with Accuracy as the evaluation objective is shown in Table 9.

**Table 9:** Performance analysis of each model on the UCI dataset.

Model	DRA + WA	DRA + ER	EBRB	GSR-BRB	DT	RF	HBRB-I
Contraceptive	0.3569	0.3641	0.4744	/	0.4932	0.5034	0.5510
Ecoli	0.8375	0.8376	0.8116	0.7826	0.8462	0.8615	0.8462
Glass	0.7026	0.6965	0.6785	0.6905	0.7143	0.6190	0.7143
Seeds	0.9214	0.9202	0.9133	0.9429	0.9048	0.9286	0.9286

As can be seen in Table 9, HBRB-I shows a good level of classification in the four datasets. Ranking first in average accuracy among BRB models, HBRB-I still shows good classification accuracy compared to machine learning models, and its interpretability makes HBRB-I model more suitable for application in high-risk domains. Meanwhile, the number of rules for each BRB model is given in Table 10. The number of rules is directly related to the amount of data since the EBRB model uses a data-driven approach to generate rules. And the excessive number of rules will lead to slow model inference, which is one of the main problems of EBRB. Unlike it, the HBRB-I model adopts a hierarchical structure to effectively limit the number of rules and exhibits good model performance. It is worth noting that if a normal BRB is used for modeling, it will lead to rule explosion because the number of rules is composed based on the Cartesian product of reference values. For example, in the Contraceptive dataset, if the traditional BRB modeling approach is used, 19,683 rules will be generated, which will greatly affect the model inference speed, so when facing more complex data, researchers often prefer to use EBRB or HBRB models for decision making.

**Table 10:** Comparison of the number of rules between different models.

Model	DRA + WA	DRA + ER	EBRB	GSR-BRB	DT	RF	HBRB-I
Contraceptive	1178	1178	1178	1178	/	/	81
Ecoli	269	269	269	269	/	/	81
Glass	172	172	172	172	/	/	81
Seeds	168	168	168	168	/	/	81

### Interpretability analysis of the model

The interpretability of the BRB model can be illustrated from two perspectives, on the one hand the IF–THEN rule-based reasoning and the clear and transparent ER inference engine of the reasoning process. In HBRB-I, which predicts global educational inequality, the 26th rule of the HBRB-I model given in Table 5 is taken as an example. The rule is shown below:

23 $$\begin{gathered} R_{26} : \, IF{\text{ (Sub - BRB - 1 }}is{\text{ High}}) \wedge (IE(2019) \, is{\text{ High)}} \wedge (IE(2020) \, is{\text{ Medium}}) \hfill \\ \quad \quad \;\;\;THEN \, \{ (\beta_{1} :0),(\beta_{2} :0),(\beta_{3} :0),(\beta_{4} :0.08),(\beta_{5} :0.3),(\beta_{6} :0.62)\} \hfill \\ \end{gathered}$$

This rule states that when the previous layer of sub-BRB outputs results in a high educational imbalance and the 2019 educational imbalance is also shown to be high and the 2020 educational imbalance is shown to be medium, the predicted 2021 educational imbalance has a probability of 0.08 to be medium, a probability of 0.3 to be highly imbalanced, and a probability of 0.62 to be very highly imbalanced. The preconditions and postconditions of this rule are basically consistent with human cognition, and thus the HBRB-I model possesses interpretability in terms of reasoning power.

Moreover, in the case of rule 26, for example, the data that activate it are often derived from data from some underdeveloped countries in Africa and South Asia. These are undoubtedly a manifestation of the interpretability of the HBRB-I model, which is consistent with the perceived causal chain, i.e., high long-run imbalances in the preconditions as well as short-run fluctuations lead to a high probability of deterioration in the regression, which is in line with the consensus of experts in the field.

**Fig. 7:**
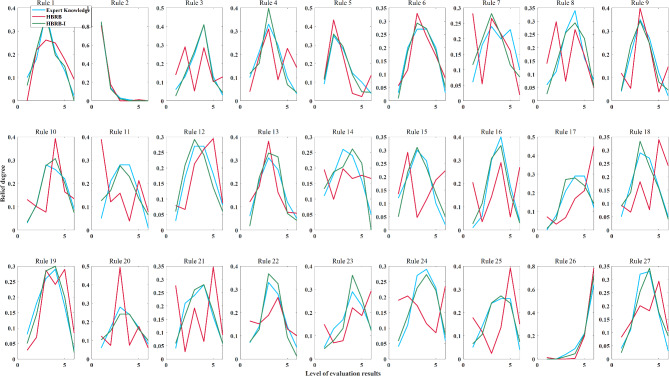
Belief degree distribution of rules for BRB models.

The interpretability of the HBRB-I model on the other hand relies heavily on reliable expert knowledge. The parameters of the BRB model reflect the experience of experts in global education analysis over a long period of time. However, the stochastic nature of the optimization algorithms often corrupts these parameters, causing them to deviate from expert knowledge or produce values that do not match human perception. Figure 7 shows the distribution of rules for the three BRB models. The HBRB-I model matches the trend of expert knowledge to some extent, indicating that the model retains the interpretability of expert knowledge. The HBRB model, on the other hand, has a large deviation from expert knowledge, indicating that it loses interpretability in the optimization process.

The interpretability of these models is particularly important for addressing global educational inequality and promoting sustainable development goals. Interpretable models provide transparency in how predictions are generated, allowing policymakers to clearly trace the underlying factors that contribute to educational inequalities. This clarity is essential for developing policies that are both equitable and sustainable, ensuring that resource allocation and interventions are based on a thorough understanding of the specific factors driving inequality. With interpretable models, projections can guide policymakers not only in addressing current educational inequalities, but also in anticipating and preventing future inequalities, thereby contributing to long-term educational sustainability. This is critical for aligning education systems with broader sustainability goals, such as those outlined in the United Nations Sustainable Development Goals (SDGs), particularly Goal 4, which aims to ensure inclusive and equitable quality education for all.

The difference between the models is that the HBRB-I model uses interpretability constraints during optimization, allowing the trained parameters to follow the same basic trends as the initial expert knowledge. In contrast, the HBRB model focuses solely on accuracy, which improves performance but loses interpretability. Analysis of the belief degree distributions in the HBRB model reveals inconsistencies, such as in rules 14, 16, and 24, where both low and high scoring attributes have high belief degrees, which defies common sense. This illustrates how traditional optimization algorithms can produce results that deviate from expert knowledge and basic human understanding.

To reflect the interpretability and readability of the model, this paper designs a quantitative metric, Expert Knowledge Fidelity (EKF), which evaluates the extent to which the model retains the a priori knowledge and logical consistency of domain experts by calculating the similarity between the rules generated by the model and the expert knowledge. This metric not only provides an objective and quantitative basis for the interpretability of the model, but also helps users understand whether the logic behind the model’s decisions is consistent with the experts’ experience. To objectively assess the consistency between model rules and expert knowledge, EKF’s computer system integrates three correlation measures, namely, cosine similarity, normalized Euclidean distance similarity and Pearson’s correlation coefficient. By analyzing the similarity from multiple perspectives, the comprehensiveness and robustness of the evaluation are improved. The specific correlation measures are calculated as follows:

24 $$\begin{gathered} sim_{\cos } = \frac{E \cdot P}{{\left\| E \right\|\left\| P \right\|}},sim_{cuc} = \frac{1}{{1 + \left\| {E - P} \right\|}},sim_{Pearson} = \frac{{{\text{cov}} (E,P)}}{{\sigma_{E} \sigma_{P} }} \hfill \\ \left\| E \right\| = \sqrt {e_{1}^{2} + e_{2}^{2} + \cdots e_{n}^{2} } ,{\text{cov}} (E,P) = \frac{1}{n}\sum\limits_{i = 1}^{n} {(e_{i} - \mu_{E} )(p_{i} - \mu_{P} )} \hfill \\ \end{gathered}$$

where $sim_{\cos } ,sim_{cuc} ,sim_{Pearson}$ denote cosine similarity, Euclidean distance similarity and Pearson correlation coefficient respectively. *E* denotes the parameter of expert knowledge, *P* denotes the model parameter, $\sigma$ denotes the standard deviation, $\mu$ denotes the mean, and *e* and *p* are the elements in *E* and *P* respectively.

For each rule the final similarity $sim_{final}$ is averaged over the three similarities mentioned above as the final similarity evaluation value. This reduces the bias of a single metric through multi-method fusion and enhances the reliability of the results. After that, a threshold $\vartheta$ is set, and the rule is judged to be consistent with expert knowledge if $sim_{final} > \vartheta$. EKF then counts the percentage of all recognized rules, denoted as:

25 $$\begin{gathered} sim_{final} = \frac{{sim_{\cos } + sim_{cuc} + sim_{Pearson} }}{3} \hfill \\ EKF = \frac{{\text{Number of rules recognized}}}{{\text{Total number of rules}}} \times 100\% \hfill \\ \end{gathered}$$

To verify the evaluation ability of expert knowledge fidelity (EKF), this experiment sets the similarity threshold to $\vartheta = 0.9$ and calculates the EKF values of the HBRB-I model and the traditional HBRB model respectively. The experimental results show (as shown in Table 11): the EKF value of the HBRB-I model is 96.30% (26/27 rules are recognized), and only the 7th rule is not recognized due to the composite similarity of 0.83 (which is slightly lower than the threshold). The EKF value of the traditional HBRB model is only 11.11% (3/27 rules are recognized), and only rules 2, 6, and 26 pass the threshold validation. The experiments illustrate that the EKF can effectively quantify the degree of inheritance of expert knowledge by the model and provide a reliable basis for the interpretability assessment; the HBRB-I model significantly improves the rule interpretability (EKF improved by 85.19%) by integrating expert constraints, which verifies the validity of this paper’s methodology; the traditional HBRB model neglects the integration of expert knowledge, and its low EKF value further highlights this paper’s the necessity of the improvement strategy.

**Table 11:** Comparative analysis of EKF values of models.

Model	EKF	Number of recognized rules	Number of non-recognized rules
HBRB	96.30%	26	1
HBRB-I	11.11%	3	24

To elucidate the core improvements of the HBRB-I model, we systematically compare the core differences between the HBRB and HBRB-I models in six dimensions: model construction, optimization algorithms, expert knowledge support, noise immunity, usage scenarios, and EKF values, as shown in Table 12. HBRB-I significantly outperforms the traditional HBRB in terms of interpretability (85.19% improvement in EKF), robustness, and scenario adaptation through a structured hierarchical design as well as an interpretable optimization algorithm. This improvement provides a trusted and intelligent support tool for high-stakes decision-making such as educational inequality governance.

**Table 12:** Comparison of HBRB and HBRB-I models.

Model	HBRB	HBRB-I
Model construction	Hierarchical structure relies on the researcher’s subjective awareness or a single layer of unstructured rule base	Self-organizing multi-layer tree structure with hierarchical handling of subproblems
Optimization algorithms	The optimization objective is more homogeneous, often minimizing only the prediction error (e.g., MSE), and is not constrained	Multi-objective optimization: prediction error as well as interpretability constraints
Degree of expert knowledge support	The optimized parameters deviate from expert knowledge and there is a risk of rules that defy physical meaning	Highly similar to expert knowledge and with practical logic, decisions are clearly visible
Noise resistance	Low (data noise tends to cause rules to deviate from actual logic)	High (interpretable constraints suppress noise interference, similarity-weighted aggregation)
Usage scenario	Coarse-grained prediction with rich data and low interpretive requirements	High-risk decision-making scenarios (e.g., policy development, medical diagnosis)
EKF value	11.11% (very low consistency of rules with expert knowledge)	96.30% (high degree of inheritance of expert knowledge)

### Analysis of global educational inequalities and recommendations for sustainable development in education

**Fig. 8:**
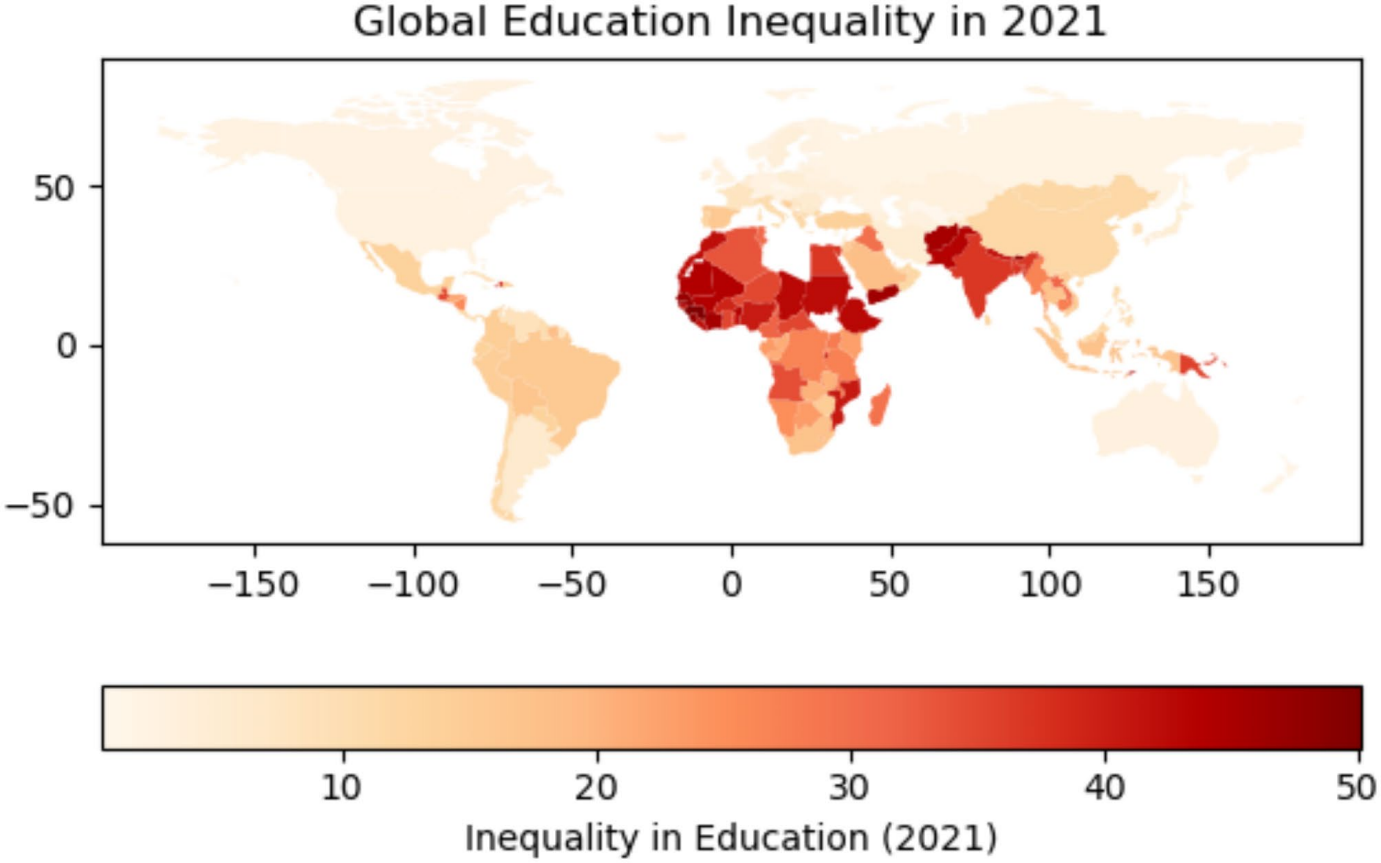
Global educational inequality distribution.

Global educational inequality has long been regarded as a key concern by governments and international organizations. It is considered not only a reflection of economic disparities but also a factor that directly affects individuals’ quality of life and future development opportunities. In many low-income countries and regions, access to the labor market and participation in sustainable social development have been restricted by the unequal distribution of educational resources and disparities in quality. A visual illustration of global educational inequality is shown in Fig. 8. Therefore, the elimination of educational inequalities is widely viewed as essential to achieving global sustainable development goals, particularly the United Nations Sustainable Development Goal 4 — ensuring inclusive, equitable, and quality education and promoting lifelong learning opportunities for all.

The HBRB-I model proposed in this study constructs an interpretable rule base to predict future education inequality risks and guide policy design by analyzing historical education inequality ratings (2014–2020) with the 2021 Human Development Index (HDI). In Section “Interpretability analysis of the model”, rule 26 is used as an example to predict that the risk level of educational inequality in South Africa and South Asia is likely to remain high in the future. Taking South Asia (SA) and Sub-Saharan Africa (SSA) as examples, the HBRB-I model captures the common mechanism of education inequality risk in the two regions: if a region’s historical education inequality rank is persistently “high” (historical inertia effect) and its current HDI is below 0.58 (development level constraint), its future The probability of a region falling into “very high risk” status in the next year exceeds 75% (Rule R26). This prediction is highly consistent with the actual data for the two regions—the average HDI in 2021 is 0.54 in SA and 0.51 in SSA, and both have a “high” education inequality rating for 2018–2020.

In South Asia (SA), The educational inequality index in South Asia remained consistently high between 2010 and 2021, with an average value of approximately 45. This suggests severe inequality in the distribution of educational resources, with little sign of significant improvement during the study period. Multiple challenges, such as poverty, a large population, and insufficient educational infrastructure, may contribute to the uneven distribution of educational resources in this region. In Sub-Saharan Africa (SSA): Sub-Saharan Africa is another region where educational inequality remains severe. Although there were slight improvements in certain years, the overall educational inequality index remained high, averaging around 34. Factors such as poverty, social instability, and a lack of public services are likely the main contributors to educational inequality in this region. Despite international aid and development projects, the issue has yet to be fundamentally addressed.

Based on the analysis of educational inequalities in different regions, this paper puts forward several recommendations to promote sustainable development in education: (1) Strengthening international assistance and cooperation: The international community should increase its support for regions with serious educational inequalities, especially in sub-Saharan Africa and South Asia, in terms of basic education and teacher training. This will help improve the educational infrastructure in these regions and ensure that all children have access to quality education. (2) Promote reforms of education systems: Countries should carry out educational reforms to improve the distribution of educational resources in accordance with their specific conditions. These reforms should aim to build a more sustainable education system in the long term, reduce inequalities, and improve the accessibility and quality of education. (3) Investing in education infrastructure: There is a need to increase investment in education infrastructure, especially in rural and remote areas, to ensure that all children have access to quality education. This will not only address current inequalities in education, but also lay a solid foundation for sustainable education in the future. (4) Promoting economic growth and social development: Economic growth and improved social conditions are essential to address the root causes of educational inequality. Economic development can provide the financial resources needed for investment in education, and social development can create a favorable environment for educational reform and make sustainable education a reality.

## Conclusions

Global educational inequality is a critical issue that not only affects socio-economic development, particularly in low-income countries and conflict-affected regions, but also undermines the sustainability of education systems worldwide. Accurately forecasting global educational inequality is essential not only to help researchers and policymakers understand the current state of education in different countries and regions, but also to provide a basis for formulating education policies and resource allocation strategies that promote long-term educational sustainability and equity. However, existing forecasting models often struggle to balance accuracy and interpretability, especially when dealing with complex data that are critical to achieving sustainable educational outcomes.

Belief rule bases (BRBs), as a type of explanatory reasoning model that integrates expert knowledge, have significant application potential. However, BRBs face several challenges in practical applications: First, due to the large amount of feature information, BRBs are prone to rule explosion during modeling. Although hierarchical BRBs can effectively mitigate this problem, a unified modeling standard is currently lacking. Second, the parameters of BRB models are typically defined by domain experts, but the stochastic nature of optimization algorithms can cause these parameters to deviate from expert knowledge, potentially compromising the interpretability of the model.

To address these issues, this paper uses an HBRB-I modeling approach based on the construction of a multilayer tree structure (MTS). In addition, this paper proposes an interpretable optimization algorithm designed for hierarchical structures and incorporating benchmark optimization. And a multi-expert knowledge consensus mechanism is proposed to ensure that the expert knowledge of the model is reliable enough. A case study using global education inequality data from the Global Human Development Report demonstrates the excellent robustness and accuracy of the HBRB-I model in predicting global education inequality, while effectively preserving the interpretability derived from expert knowledge. These results validate both the reliability of the model and the effectiveness of the proposed optimization algorithm. The HBRB-I model provides a transparent decision-making process that enables efficient forecasting, particularly in support of equitable resource allocation and policy formulation in education. This interpretability plays a crucial role in promoting educational equity and advancing the achievement of the UN Sustainable Development Goals, particularly SDG 4—ensuring inclusive and equitable quality education for all.

However, the research in this paper still has some limitations. Although the multi-expert consensus mechanism can balance the individual cognitive differences by weighted aggregation of multi-expert knowledge and thus make the expert knowledge of the model reliable enough. However, if different experts may give contradictory belief degree for the same rule, this may lead to the rule belief degree deviating from the true law. In addition, although the MTS-based construction method can self-organize to achieve the modeling process, the size of each internal node determines the overall construction process, and the size of the internal nodes is often judged by the researcher on his/her own according to the actual situation. In future research, researchers can compensate for the lack of expert knowledge by using a data-driven approach, and at the same time adopt the inclusion of internal node sizes in data-driven as well to realize the construction of automated HBRB.

## Data Availability

The datasets analysed during the current study are available in the following link: https://www.kaggle.com/datasets/iamsouravbanerjee/inequality-in-education-around-the-world.
